# Metformin and Dietary Restriction Counteract Aging via Reducing m6A–Dependent Stabilization of Methionine Synthase mRNA in *Brachionus asplanchnoidis* (Rotifera)

**DOI:** 10.1111/acel.70113

**Published:** 2025-05-27

**Authors:** Yu Zhang, Xiaojie Liu, Hairong Lian, Yanchao Chai, Yang Zhou, Dongqi Kan, Jilong Ren, Cui Han, Jiaxin Yang

**Affiliations:** ^1^ College of Marine Science and Engineering Nanjing Normal University Nanjing Jiangsu People's Republic of China; ^2^ Jiangsu Province Key Laboratory of Live Food for Fisheries Nanjing China; ^3^ School of Ecology and Environment Anhui Normal University Wuhu Anhui People's Republic of China

**Keywords:** antiaging, dietary restriction, m6A, metformin, methionine metabolism, MTR, rotifer

## Abstract

Metformin, a medication primarily used to treat diabetes, has gained attentions for its potential antiaging properties. Although the metabolic and cellular pathways behind its longevity effects have been widely studied, few studies have explored the epigenetic regulatory effects of metformin, which are a crucial factor in aging processes. In this study, we examined the antiaging effects of metformin using the *Brachionus* rotifer as a model, focusing on the regulation of mRNA N6–methyladenosine (m6A), a key RNA modification involved in mRNA stability, translation, and splicing. We found metformin significantly extended the rotifers' lifespan, mimicking the effects of dietary restriction (DR), a well–established antiaging intervention. Both metformin and DR modulate m6A dynamics, with a notable reduction in the m6A modification of MTR (5–methyltetrahydrofolate–homocysteine methyltransferase). This reduction led to decreased MTR expression and lowered levels of S–adenosylmethionine (SAM), a critical metabolite in the one–carbon cycle. We propose that the downregulation of MTR through m6A modification limits methionine synthesis and imposes methionine restriction, a key factor in promoting longevity. Our findings reveal a novel epitranscriptional regulatory model by which metformin and DR modulate m6A to extend lifespan, highlighting MTR as a central regulator of aging and suggesting potential therapeutic strategies for healthy aging through m6A and methionine metabolism.

## Graphical abstract

Metformin and dietary restriction modulate m6A to extend lifespan of rotifer, highlighting MTR as a central regulator of aging through methionine metabolism.

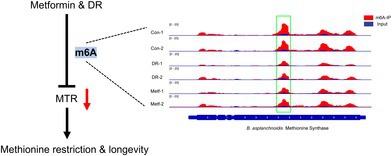

## 1 Introduction

Aging is an inevitable and progressive process that leads to physiological dysfunction and tissue deterioration, yet it can be modulated artificially by various interventions, including dietary restriction (DR), genetic manipulation, or the use of small molecule drugs (Kenyon et al. 1993; Partridge et al. 2020; Yalcin and Lee 2020). Numerous studies have introduced drugs with antiaging effects, including acarbose, spermidine, rapamycin, metformin, etc., which have been well reviewed by others (Partridge et al. 2020; Yalcin and Lee 2020). Notably, metformin, a classic FDA–approved antidiabetes drug, has attracted attention for its potential to extend lifespan and promote healthy aging in multiple model organisms and humans (Cabreiro et al. 2013; Martin‐Montalvo et al. 2013; Onken and Driscoll 2010; Snell and Johnston 2014).

Mechanistically, metformin accumulates in mitochondria, where it specifically inhibits mitochondrial complex I. This inhibition raises the cytoplasmic AMP: ATP ratios, leading to the phosphorylation and activation of AMPK, an essential energy sensor that maintains energy homeostasis and regulates glucose and fat metabolism (Carling et al. 2011; El‐Mir et al. 2000; Zhang et al. 2009). This process results in adjusted nutrient sensing (Nadal et al. 2013), enhanced protein homeostasis (proteostasis) (Wang et al. 2017), improved mitochondrial function (Aatsinki et al. 2014), and maintained genomic stability (Algire et al. 2012; Vazquez‐Martin et al. 2011). Additionally, metformin reduces cellular inflammation and alters intercellular communication through AMPK‐independent pathways (Cameron et al. 2016). Although these metabolic and cellular pathways have been widely studied, the role of metformin in epigenetic regulation, a crucial factor in aging processes, remains poorly understood.

Epigenetic modifications, including the acetylation and methylation of histones or DNA, are known to play essential roles in aging by influencing chromatin structure and gene transcription (Michalak et al. 2019; Pal and Tyler 2016; Sen et al. 2016). Metformin has been shown to modulate such modifications, altering gene expression through histone and DNA methylation. Additionally, metformin has been implicated in the regulation of noncoding RNAs and microRNAs, further expanding its influence on gene expression (Bridgeman et al. 2018; Cuyas et al. 2018; Cuyàs et al. 2018a, 2018b; Noren Hooten et al. 2016; Zhong et al. 2017). Emerging evidence suggests that RNA modifications, particularly N6‐methyladenosine (m6A), are key regulators of aging processes (Sun et al. 2022; Wu et al. 2023; Zhang and Xia 2023). m6A is the most prevalent and evolutionarily conserved RNA modification in eukaryotic mRNA, involved in various biological functions, including mRNA stability, splicing, and translation (Lee et al. 2014; Meyer and Jaffrey 2017; Zaccara et al. 2019). Given its crucial role in regulating mRNA fate, m6A is linked to age‐related diseases and cellular aging processes (Huang et al. 2023; Jiang et al. 2021; Petrosino et al. 2022). Despite growing interest in m6A's role in aging, whether metformin or DR directly influences m6A modifications to exert its antiaging effects remains largely unknown.

Short‐lived animal models are of great value in geroscience and experimental gerontology (Piper and Partridge 2018; Tissenbaum 2014; Wilkinson et al. 2012). In this study, we employed the monogonont rotifer *Brachionus asplanchnoidis*, a well‐established model in aging research, which is amenable to genetic manipulation and can reproduce via parthenogenesis in the laboratory, allowing experiments to be conducted on a consistent genetic background without the need to develop highly inbred strains (Enesco 1993; Feng et al. 2023; Gribble and Mark Welch 2017; Kaneko and Yoshinaga 2017; King 1969; Lansing 1947, 1948; Shearer and Snell 2007; Snell 2014; Snell et al. 2015; Zhang et al. 2024). Additionally, rotifers belong to the superphylum Lophotrochozoa, part of Protostomia, and have not undergone extensive genome reduction (Kortschak et al. 2003; Takahashi et al. 2009; Wyder et al. 2007). They possess many vertebrate gene homologs that are absent in *Drosophila melanogaster* and *Caenorhabditis elegans* (Gribble and Mark Welch 2017). A previous study has shown that rotifers possess a functional m6A methylation system, making them uniquely suited to study m6A‐regulated epitranscriptional aging mechanisms, unlike *C. elegans* , which is deficient in mRNA m6A modification (Sendinc et al. 2020; Zhang et al. 2023).

In this study, we treated *B. asplanchnoidis* rotifers with metformin and DR to investigate their impact on m6A and the aging process. Both metformin and DR are widely studied antiaging interventions. Since metformin is often considered a DR mimic, it is plausible that they may share similar epigenetic mechanisms involving m6A modifications. We found metformin significantly delays the aging of rotifers, reduces aging‐related biomarkers, and slows down the reproductive clock, mirroring the effects of DR. However, metformin does not further extend the lifespan of rotifers beyond what is achieved by DR alone. Using joint analysis of m6A‐MeRIP‐seq (m6A methylated RNA immunoprecipitation sequencing) and mRNA‐seq, we found that both metformin and DR can regulate m6A dynamics, affecting gene transcription‐related processes. Among them, MTR (5‐methyltetrahydrofolate‐homocysteine methyltransferase) emerged as a key m6A effector. Both metformin and DR significantly downregulated the m6A modification of MTR, diminishing the IGF2BP (Insulin‐like growth factor 2 mRNA binding protein)‐mediated mRNA stabilization effect and accelerating MTR mRNA degradation. Further investigation revealed that mild knockdown of MTR mimics the phenotypes induced by metformin and DR in rotifers. We propose metformin and DR reduce MTR transcript stability via the m6A pathway, reducing MTR expression and subsequently methionine resynthesis, which imposes methionine restriction. In conclusion, our findings suggest a potential epitranscriptional mechanism by which metformin and DR exert antiaging effects through m6A modifications. Specifically, we identify MTR as a central epigenetic regulator mediating these interventions, highlighting a potential strategy to promote healthy longevity through targeted m6A and methionine metabolism modulation.

## 2 Results

### 2.1 Metformin Extends Lifespan and Induces a DR‐Like Phenotype in Rotifers

**FIGURE 1:**
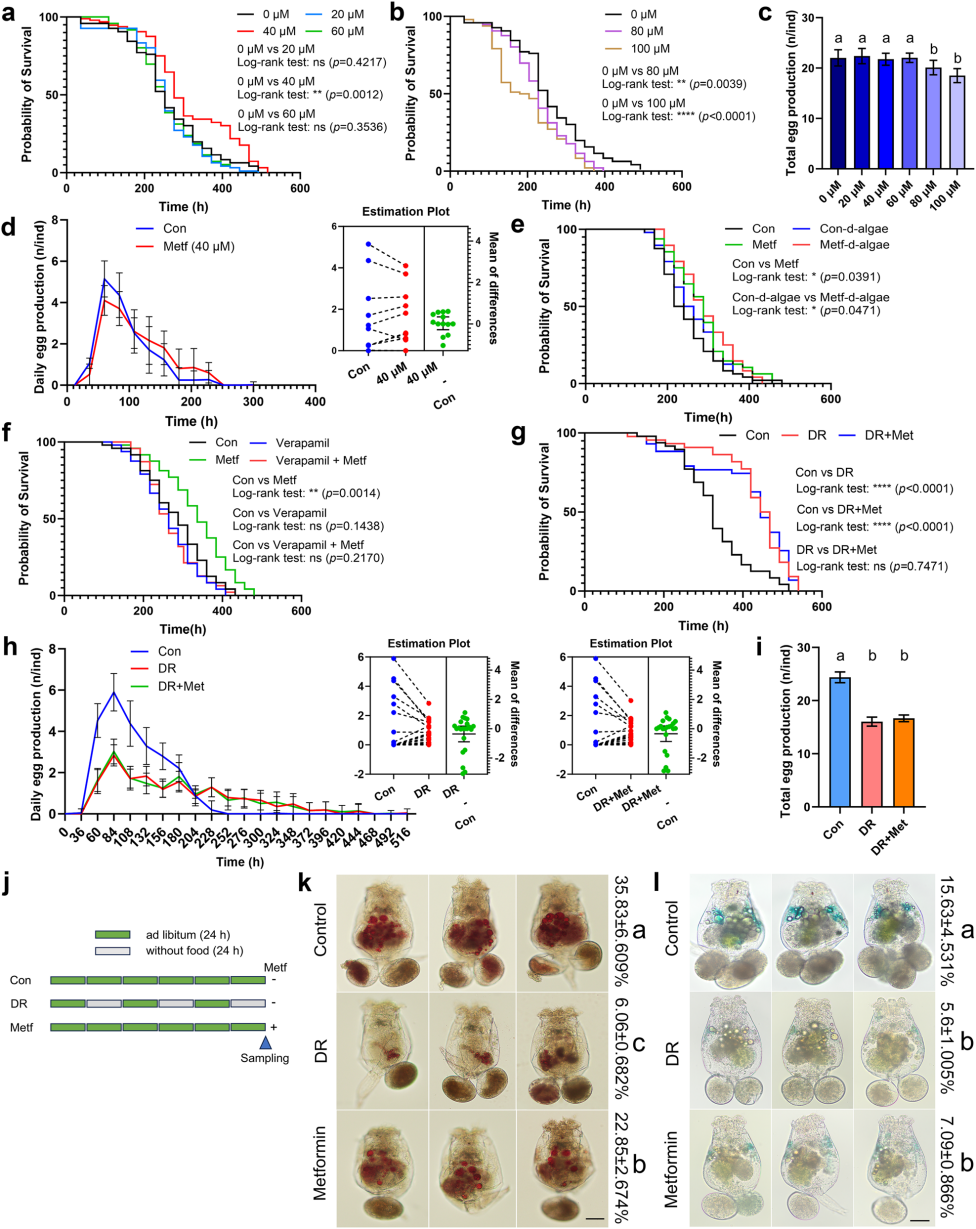
Metformin significantly extends the lifespan of rotifers and shares some phenotypes with DR. (a, b) Survival curve analysis of rotifers exposed to 0 μM, 20 μM, 40 μM, 60 μM, 80 μM and 100 μM metformin. While 40 μM metformin significantly extends rotifer lifespan, higher concentrations (80 μM, 100 μM) reduce survival due to toxicity. Intermediate concentrations (20 μM, 60 μM) have no significant effect compared to the control. (c) Comparison of total egg production of rotifers treated with different concentrations of metformin. 40 μM metformin does not affect the lifetime egg laying capacity. Data are presented as mean ± SD, *n* = 48. Kruskal–Wallis test, *p* < 0.0001. Dunn post hoc, a > b, *α* = 0.05. (d) Comparison of daily egg production between control and 40 μM metformin‐treated group. The differences are presented by the estimation plot, and the upper and lower limits on the right side are 95% confidence intervals. (e) Metformin continues to extend the lifespan of rotifers even when their food is inactivated. Algae are inactivated through quick‐freezing with liquid nitrogen. (f) Inhibiting the transport of metformin by verapamil (15 μM) abolishes the life‐extending effect of metformin on rotifers. (g) Survival curve analysis of rotifers treated with DR or both with metformin. Metformin cannot further extend the lifespan of the rotifers that have undergone DR. (h) Comparison of daily egg production between control, DR, and cotreatment. The estimation plots show the specific differences, with the 95% confidence intervals shown on the right side of each plot. (i) Comparison of total egg production of rotifers between control, DR, and cotreatment. Data are presented as mean ± SD, *n* = 48. Kruskal–Wallis test, *p* < 0.0001. Dunn post hoc, a > b, *α* = 0.05. (j) Schematic diagram showing DR and metformin treatment, and the sampling time (6d) for rotifers used in staining. (k) Both metformin and DR reduce lipid content in rotifers. Data are presented as mean ± SD, *n* = 3. *F*_2,6_ = 39.07, *p* = 0.0004. Tukey post hoc, a > b > c, *α* = 0.05. Scale bar, 50 μm. (l) Both metformin and DR reduce SA‐β‐Gal activity in rotifers. Data are presented as mean ± SD, *n* = 3. *F*_2,6_ = 11.82, *p* = 0.0083. Tukey post hoc, a > b, *α* = 0.05. Scale bar, 50 μm.

Given the inconsistent performance of metformin across different animal models, we first evaluated its impact on rotifers. Rotifers continuously exposed to 40 μM metformin exhibited a significantly extended lifespan, with the median lifespan increased by 9.52%, accompanied by a right‐shifted survival curve. However, higher concentrations of metformin proved toxic to the rotifers and reduced the lifetime egg laying capacity (Figure 1a–c). Furthermore, although 40 μM metformin did not affect total egg production, the reproductive clock was significantly slowed down, suggesting that the energy distribution may be regulated by metformin (Figure 1d).

Based on previous report that metformin can indirectly affect the metabolism of nematodes by influencing the metabolism of the bacteria they consume as food (Cabreiro et al. 2013), we deactivated the food source of rotifers (algae) and reevaluated the survival of the rotifers. We found that metformin still extended the lifespan of rotifers (Figure 1e). In addition, we used verapamil to block OCT1 (Organic Cation Transporter 1), the transport protein responsible for metformin uptake (Cho et al. 2014; Gong et al. 2012), and found that it abolished the life‐prolonging effect of metformin on rotifers (Figure 1f). These results suggest that metformin acts directly on rotifers to extend their lifespan.

Dietary restriction, which extends lifespan by reducing food intake without malnutrition, is often used as a benchmark for evaluating other longevity‐enhancing interventions (Forster 2003; Mair et al. 2005). In our study, we employed an intermittent fasting approach to implement DR in rotifers (Gribble and Welch 2013). We observed that DR significantly prolonged the lifespan, reduced lifetime egg production, slowed the reproductive clock, and decreased lipid content and senescence‐associated beta‐galactosidase (SA‐β‐Gal) activity in rotifers (Figure 1g–l). Surprisingly, metformin did not extend additional lifespan when administered in combination with DR but caused some side effects, hastening the death of rotifers in the early life stage (Figure 1g). Intriguingly, metformin mimicked some symptoms with DR, slowing down the reproductive clock, reducing lipid deposit, and SA‐β‐Gal activity (Figure 1d,h,k,l). In summary, metformin directly acts on rotifers to delay aging and induces DR‐like phenotypes, implying that metformin may share underlying molecular mechanisms with DR in rotifers.

### 2.2 m6A Decrease and Remodeling Accompanies Metformin Administration or DR

**FIGURE 2:**
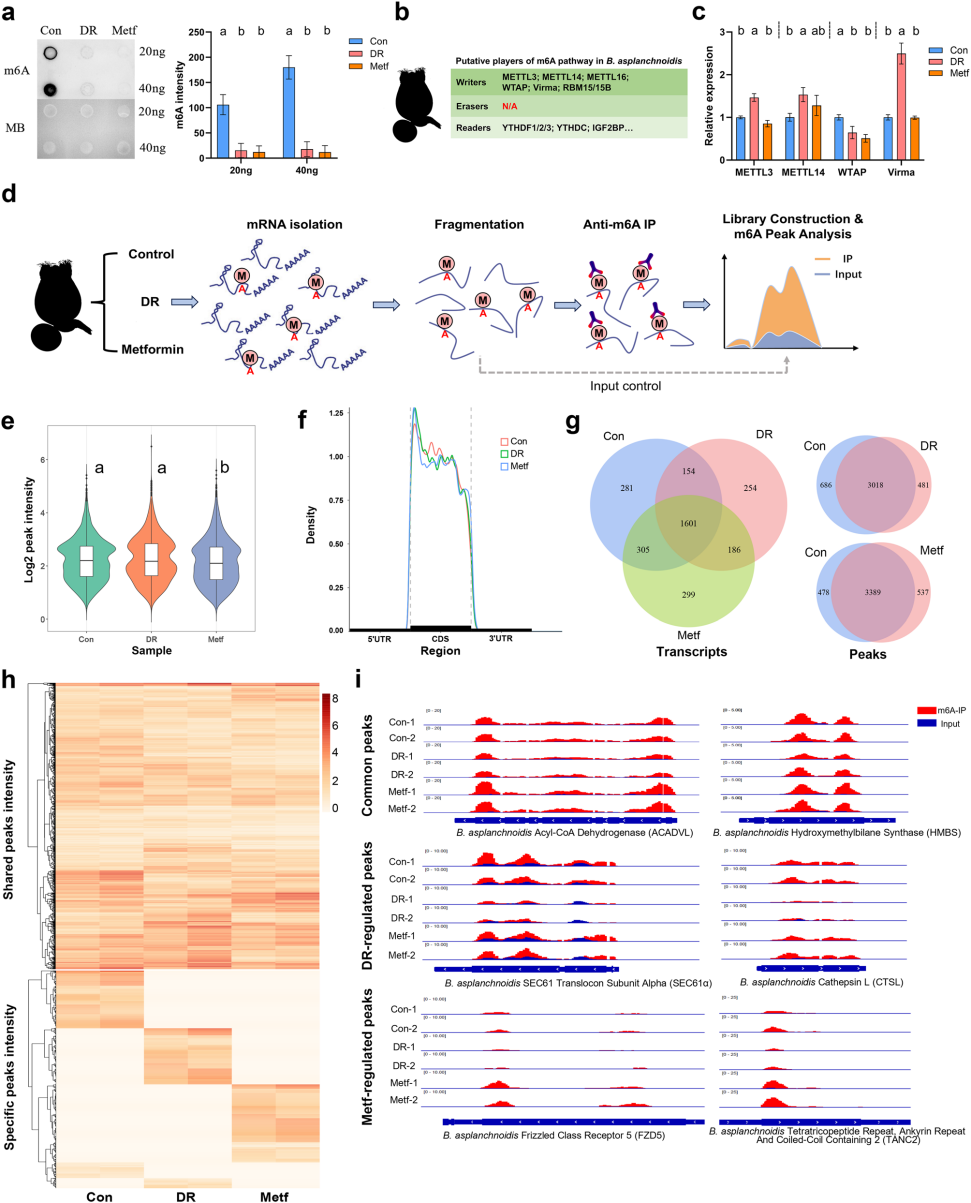
Metformin and DR reduce mRNA m6A levels and alter the m6A profile in rotifers. (a) Dot blot analysis of mRNA m6A levels in rotifers was conducted among the control, DR and metformin groups. Methylene blue (MB) staining served as RNA loading control. Both DR and metformin treatment reduced mRNA m6A levels. Data are presented as mean ± SD, *n* = 3. 20 ng: *F*_2,6_ = 34.58, *p* = 0.0005; 40 ng: *F*_2,6_ = 88.52, *p* < 0.0001. Tukey post hoc, a > b, *α* = 0.05. (b) Summary of putative players of m6A in rotifers. m6A demethylases are found in vertebrates and absent in rotifers. (c) Relative expression of core members of the m6A transferase complex was assessed. Both DR and metformin treatment reduced the relative expression of WTAP. Data are presented as mean ± SD, *n* = 3. METTL3: *F*_2,6_ = 62.08, *p* < 0.0001; METTL14: *F*_2,6_ = 6.969, *p* = 0.0273; WTAP: *F*_2,6_ = 17.79, *p* = 0.003; Virma: *F*_2,6_ = 102.7, *p* < 0.0001. Tukey post hoc, a > b, *α* = 0.05. (d) Schematic diagram of m6A‐MeRIP‐seq workflow. Isolated mRNA was chemically fragmented into ∼100‐nucleotide fragments and immunoprecipitated using an m6A‐specific antibody. Eluted RNA from immunoprecipitation and input control RNA were used for library construction and sequencing. (e) Violin and box plots depicting the overall m6A methylation intensity in rotifers for the control, DR and metformin groups. Horizontal lines show mean intensity, boxes indicate 25th and 75th percentiles, and dots show outliers. Kruskal–Wallis test, *p* < 0.0001. Dunn post hoc, a > b, *α* = 0.05. (f) Peak density plot showing the m6A density and distribution on mRNA, with most m6A peaks located in coding regions and a small amount of m6A modification near the 3′ UTR. The m6A signal at the 5′ end of CDS might be associated with m6Am modification, which as reported, can also be recognized by m6A‐specific antibodies. (g) Venn diagram illustrating the m6A‐modified transcripts and m6A peaks among the control, DR, and metformin‐treated groups. (h) Clustered heatmap of the m6A intensity shared among the control, DR, and metformin groups, as well as the m6A intensity specifically regulated in each group. The color key from light to dark represents m6A enrichment from low to high. (i) IGV plots showing examples of shared m6A peaks, as well as m6A peaks specifically regulated by DR and metformin treatment.

To determine the role of m6A to metformin‐ or DR‐induced longevity, we first examined the m6A levels on mRNA and the transcriptional levels of core components involved in m6A “writer” complex (m6A methyltransferases) from rotifers treated with metformin or DR. We found that m6A levels and the expression of WTAP (Wilm's Tumor‐1 Associated Protein) were dramatically reduced in both metformin‐ and DR‐treated rotifers (Figure 2a–c). WTAP is essential for the accumulation of the METTL3‐METTL14 heterodimer in nuclear speckles (Ping et al. 2014; Scholler et al. 2018). Next, we performed m6A‐MeRIP‐seq to map the transcriptome‐wide m6A landscape in rotifers (Figure 2d). This approach provided insights into the identity of m6A‐modified mRNAs. We detected approximately 4000 m6A peaks in *B. asplanchnoidis*, with m6A modifications predominantly found in coding regions, showing subtle differences in peak density and frequency across control, metformin, and DR treatments (Figure 2e,f & Figure S1). Further analysis revealed that m6A modifications were typically located in a consensus “AGACY” motif (Y: U/C), which is similar to the “RRACH” motif (R: A/G; H: U/A/C) reported in vertebrates (Figure S2). While a core set of m6A peaks on the transcriptome remained unchanged, our analysis revealed that the m6A landscape was remodeled in metformin‐treated rotifers, with 537 peaks gained on 485 new mRNAs and 478 peaks lost by 435 mRNAs. Similarly, rotifers subjected to DR also displayed significant changes in m6A modification, with 440 new transcripts acquiring 481 peaks and 686 peaks being lost from 586 mRNAs (Figure 2g). Further analysis revealed that each treatment specifically regulated hundreds of m6A peaks, with DR modulating 447 peaks and metformin affecting 713 peaks (Figure 2h,i & Data S1 and S2). Transcripts with shared or specific m6A peaks were enriched in many key biological processes, such as the regulation of transcription, development, signal transduction, mRNA processing, and splicing. Notably, the transcription regulation process was particularly widely enriched among these transcripts (Figures S3–S6). In summary, our findings indicate that m6A methylation is prevalent in rotifers and can be modulated by both metformin and DR.

### 2.3 MTR Functions as a Key m6A Target Regulated by Metformin and DR

**FIGURE 3:**
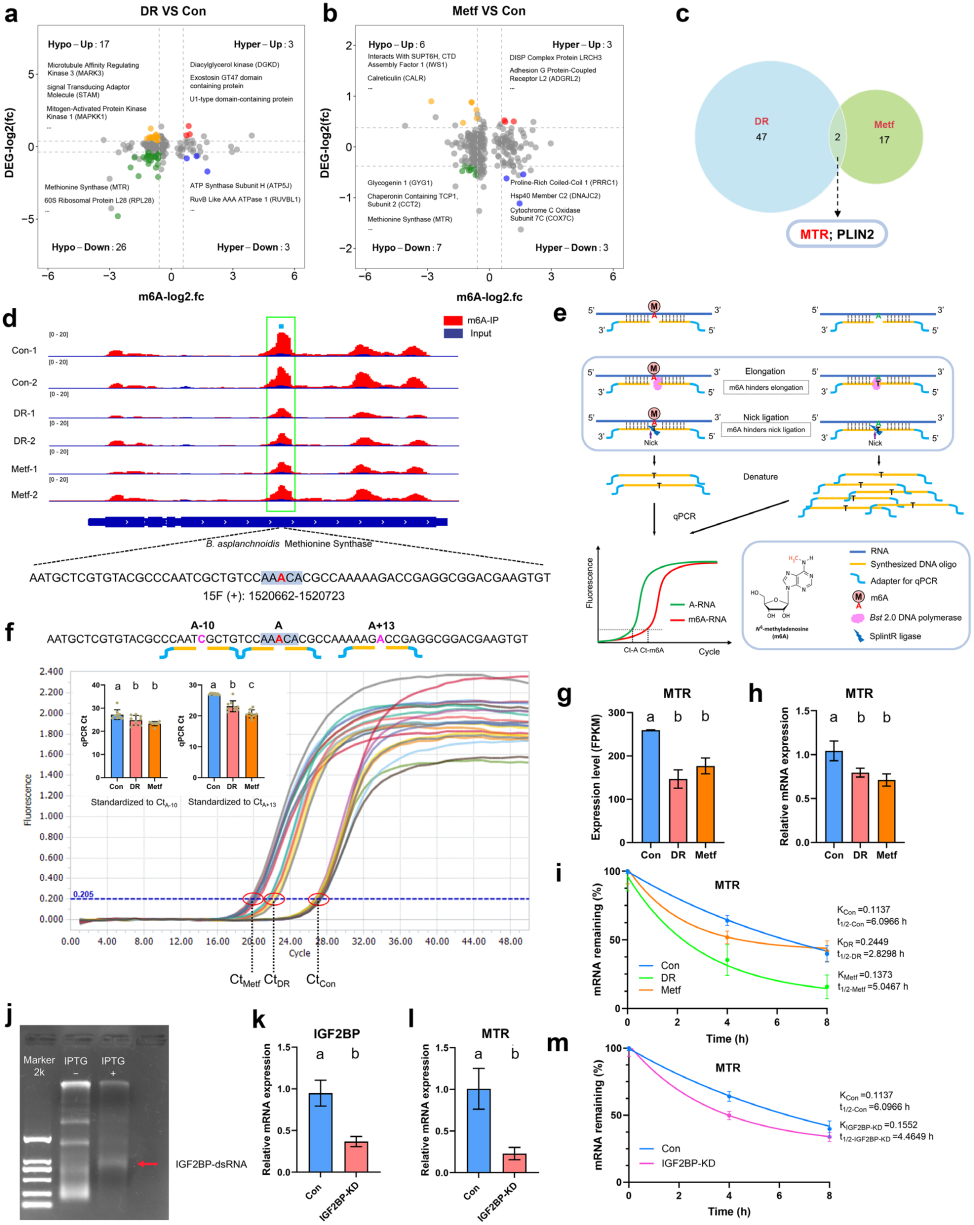
Metformin and DR reduce MTR expression by attenuating m6A levels and m6A‐mediated stability. (a, b) Joint analysis of changed m6A levels and differential gene expression in DR or metformin‐treated rotifers. m6A: |Fold‐change| ≥ 1.5, *p* ≤ 0.05; DEGs: |Fold‐change| ≥ 1.3, *p* ≤ 0.05. Hyper‐up: M6A up & expression up; Hyper‐down: M6A up & expression down; Hypo‐up: M6A down & expression up; Hypo‐down: M6A down & expression down. (c) Venn diagram showing the 2 genes coregulated by DR and metformin. (d) IGV plot indicates that m6A modifications on MTR mRNA were reduced in both DR and metformin‐treated rotifers, and the differential modification site is highlighted. (e, f) Schematic diagram of the “SELECT” workflow, and the workflow was used to validate the changes in m6A of MTR. “A + 13” and “A‐10” are the sites upstream and downstream of the modification site, respectively, used to correct the input RNA quantity. Data are presented as mean ± SD, *n* = 9. “A + 13”: *F*_2,24_ = 58.47, *p* < 0.0001; “A‐10”: *F*_2,24_ = 10.91, *p* = 0.0004. Tukey post hoc, a > b > c, *α* = 0.05. (g) Relative abundance of MTR (FPKM) in control, DR‐, and metformin‐treated rotifers. Data are presented as mean ± SD, *n* = 2. *F*_2,3_ = 26.41, *p* = 0.0125. Tukey post hoc, a > b, *α* = 0.05. (h) qPCR analysis of MTR mRNA levels in control, DR‐, and metformin‐treated rotifers. Data are presented as mean ± SD, *n* = 3. *F*_2,6_ = 13.34, *p* = 0.0062. Tukey post hoc, a > b, *α* = 0.05. (i) The MTR stability is reduced in DR‐ and metformin‐treated rotifers. (j) Induction of IGF2BP dsRNA via in vivo transcription. The indicated band represents the dsRNA. (k) Assessing the knockdown efficiency of IGF2BP using qPCR analysis. Data are presented as mean ± SD, *n* = 3. *t* = 6.04, df = 4, *p =* 0.0038 (two‐tailed), a > b, *α* = 0.05. (l) Expression of MTR in IGF2BP‐deficient rotifers. Data are presented as mean ± SD, *n* = 3. *t* = 5.26, df = 4, *p =* 0.0063 (two‐tailed), a > b, *α* = 0.05. (m) The MTR stability is reduced in IGF2BP‐deficient rotifers.

We next profiled the m6A epitranscriptome in rotifers treated with metformin and those subjected to DR. Functional category analysis of differentially peaked transcripts also revealed a stable presence of m6A on mRNAs associated with transcription regulators and mitochondrial functions, including transcription by RNA polymerase II, and the mitochondrial proton‐transporting ATP synthase complex (Figure S7). These data suggest potential crosstalk between m6A‐dependent posttranscriptional gene regulation and transcriptional processes. Subsequently, we conducted mRNA sequencing (mRNA‐seq) and integrated the differentially expressed genes (DEGs) with m6A‐MeRIP‐seq analysis, identifying MTR (5‐methyltetrahydrofolate‐homocysteine methyltransferase), also known as methionine synthase, which encodes an important regulator of one‐carbon metabolism (Figure 3a–c). The m6A modification in MTR was markedly reduced in both metformin‐ and DR‐treated rotifers, as validated by the “SELECT” method (single‐base elongation and ligation‐based qPCR amplification) (Figure 3d–f). We also observed a significant decrease of MTR expression at the mRNA level in rotifers administered metformin as well as those that underwent DR (Figure 3g,h).

The reduction in m6A modification levels and the decrease in MTR transcript abundance indicate that the stability of MTR transcripts may be modulated by m6A‐dependent regulation. Then we measured the amounts of remaining mRNAs after actinomycin D (ACTD) was used to treat rotifers. Consistent with expectations, we observed a shortened mRNA half‐life of MTR (t_1/2_, 50% of original abundance) in metformin and DR‐treated rotifers, from 6.1 h to 5.05 h and 2.83 h, respectively (Figure 3i). Previous studies have shown that IGF2BPs (Insulin‐like growth factor 2 mRNA binding proteins) are responsible for m6A recognition and the m6A‐dependent regulation of mRNA stability (Huang et al. 2018; Xu et al. 2025). Therefore, we investigated the potential interaction between IGF2BP and MTR mRNA in rotifers. As expected, we also observed a significant decrease in the MTR half‐life and mRNA expression in IGF2BP‐deficiency rotifers (Figure 3j–m). This suggests that IGF2BP may account for the regulation of MTR mRNA stability. Altogether, these results suggest that MTR is a critical effector in the m6A pathway regulated by metformin or DR.

### 2.4 Mild Downregulation of MTR Delays Aging in Rotifers

**FIGURE 4:**
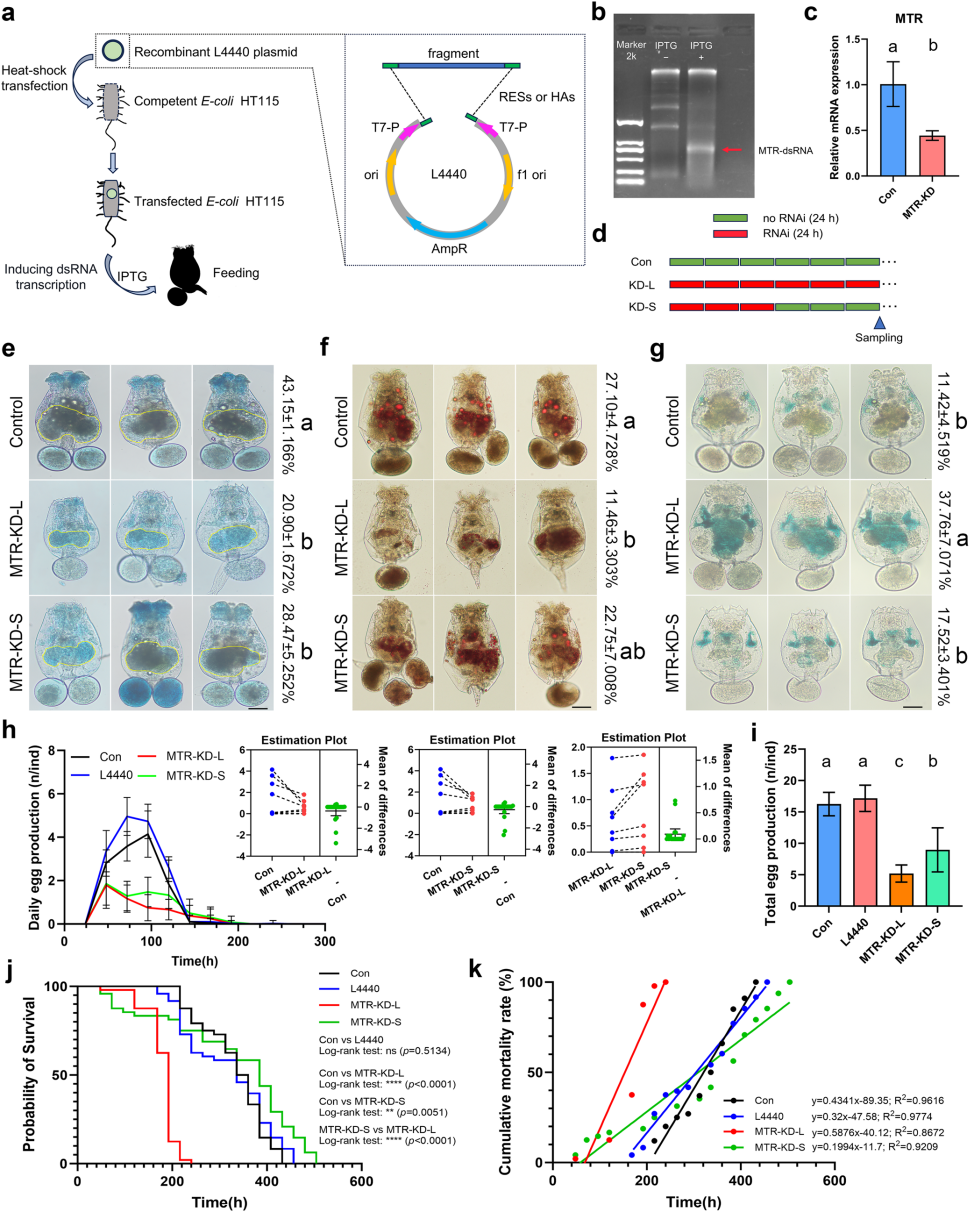
A gentle decrease of MTR delays aging in rotifers. (a) Schematic diagram of feeding‐RNAi workflow of the rotifer. RESs represent Restriction Enzyme Sites, and HAs represent Homology Arms. (b) Induction of MTR dsRNA via in vivo transcription. The indicated band represents the dsRNA. (c) Assessing the knockdown efficiency of MTR using qPCR analysis. Data are presented as mean ± SD, *n* = 3. *t* = 3.894, df = 4, *p =* 0.0176 (two‐tailed), a > b, *α* = 0.05. (d) Schematic diagram of the treatment with long‐term knockdown (KD‐L) and short‐term knockdown (KD‐S). (e) Knockdown of MTR hindered the development of the rotifers' ovary. Data are presented as mean ± SD, *n* = 3. *F*_2,6_ = 36.29, *p* = 0.0004. Tukey post hoc, a > b, *α* = 0.05. (f) MTR deficiency reduced the lipid storage in rotifers. Data are presented as mean ± SD, *n* = 3. *F*_2,6_ = 7.114, *p* = 0.0261. Tukey post hoc, a > b, *α* = 0.05. (g) Short‐term knockdown of MTR does not increase the activity of SA‐β‐Gal. Data are presented as mean ± SD, *n* = 3. *F*_2,6_ = 20.87, *p* = 0.002. Tukey post hoc, a > b, *α* = 0.05. (h) Comparison of daily egg production of rotifers between the blank control, the positive control (L4440, empty plasmid), MTR long‐term knockdown, and MTR short‐term knockdown groups. MTR deficiency reduced daily egg production and extended the reproductive period. The estimation plots show the specific differences, with the 95% confidence intervals shown on the right side of each plot. (i) MTR deficiency reduced the lifetime egg production. Data are presented as mean ± SD, *n* = 48. Kruskal–Wallis test, *p* < 0.0001. Dunn post hoc, a > b > c, *α* = 0.05. (j, k) Survival curve and cumulative mortality rates analyses in rotifers with blank control, positive control (L4440, empty plasmid), long‐term knockdown of MTR, and short‐term knockdown of MTR. The short‐term knockdown of MTR reduced the mortality rate in the late life stage of the rotifer.

To investigate whether the downregulation of MTR plays a key role in the antiaging effects mediated by metformin and DR, we generated MTR‐deficient rotifers using a feeding‐RNAi strategy (Figure 4a–d). Long‐term knockdown of MTR resulted in severe developmental disorders, with ovaries approximately 21% smaller than those in the control group (Figure 4e). Correspondingly, there was a significant decrease in egg laying capacity and lipid content (Figure 4f,i). Additionally, we detected an increase in SA‐β‐Gal activity in MTR‐deficient rotifers (Figure 4g). These results contradicted our initial expectations, prompting us to question whether they were due to excessive interference with MTR, as metformin and DR only moderately downregulate this gene. We then conducted a short‐term RNAi of MTR in rotifers, followed by a recovery period (Figure 4d). During the first 3 days, the vitality of the rotifers was poor, with significantly lower daily egg production compared to the control group, and they consumed very little food. In the subsequent recovery phase, the egg laying capacity of the rotifers partially recovered but remained lower than that of the control group, and the reproductive period was significantly extended, similar to that in rotifers treated with DR or metformin (Figure 4h,i). As for the survival of the rotifers, the mortality rate was higher in the early life stage compared to the control group but significantly lower in the later stage (Figure 4j,k). Taken together, these findings suggest that MTR is essential for maintaining the health of rotifers, and moderate downregulation is beneficial for delaying aging.

### 2.5 Inhibiting SAM Production Extends the Lifespan of Rotifers

**FIGURE 5:**
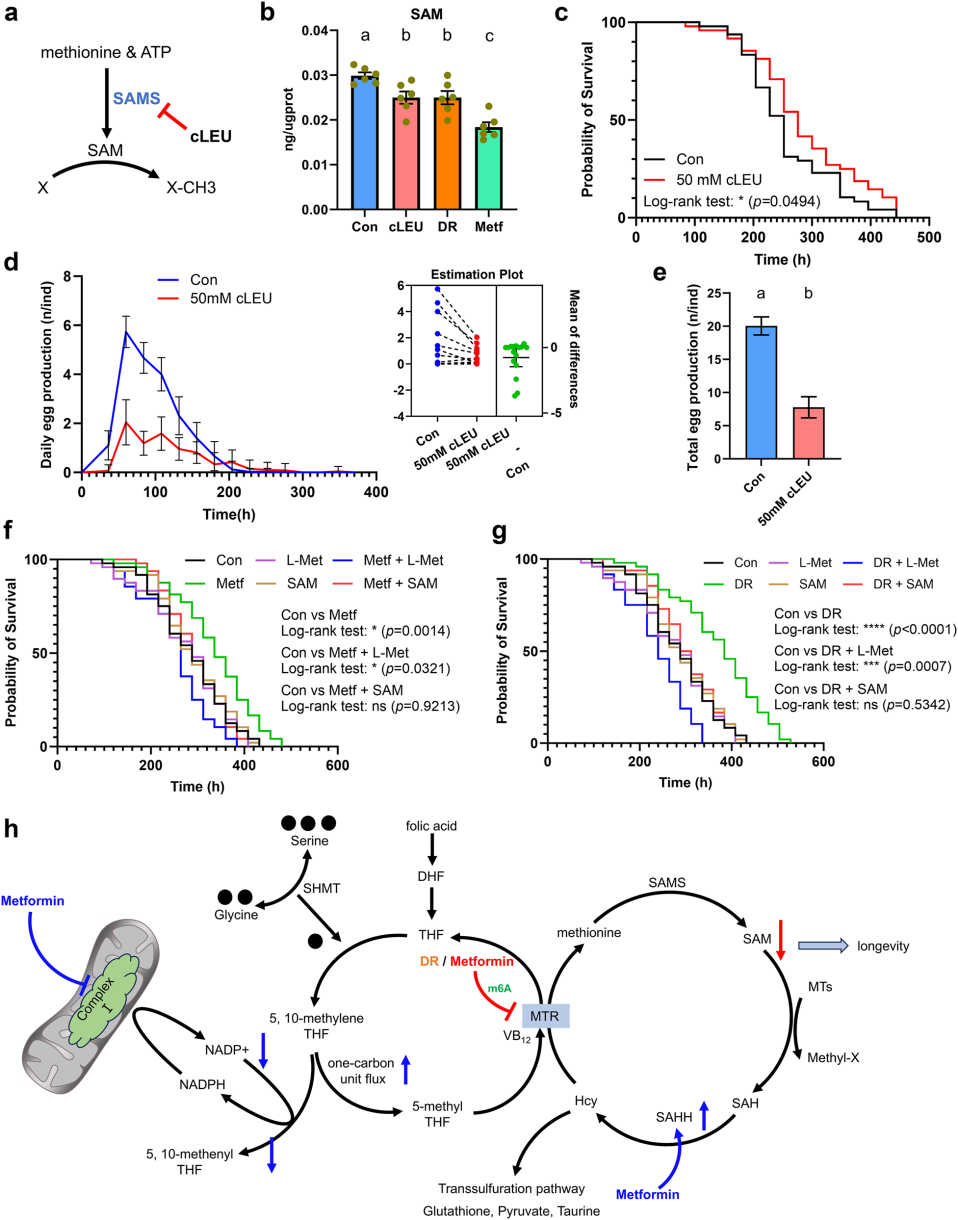
Inhibiting methionine metabolism extends the lifespan of rotifers. (a) Schematic diagram of cycloleucine (cLEU) inhibiting methionine metabolism. (b) Metformin and DR reduce SAM. Data are presented as mean ± SD, *n* = 6. *F*_3,20_ = 15.32, *p* < 0.0001. Tukey post hoc, a > b > c, *α* = 0.05. (c) Survival curve analysis of rotifers treated with 50 mM cLEU. cLEU extends lifespan of the rotifers. (d) Comparison of daily egg production between control and cLEU‐treated group. The estimation plots show the specific differences and the 95% confidence interval is shown on the right. (e) cLEU reduces the total egg production of rotifers. Data are presented as mean ± SD, *n* = 48. *t* = 40.37, df = 94, *p <* 0.0001 (two‐tailed), a > b, *α* = 0.05. (f, g) Excessive supplementation of methionine (1 mM) or SAM (20 μM) hindered the life‐prolonging effect of metformin and DR. (h) A model illustrating the targets and negative regulation mechanisms of metformin and DR in one‐carbon metabolism. The blue lines and arrows indicate the reported pathways of action of metformin, while the red lines and arrows represent metformin's pathways of action proposed in this study. “●” means a one‐carbon unit; DHF, dihydrofolate; Hcy, homocysteine; MTR, 5‐methyltetrahydrofolate‐homocysteine methyltransferase; MTs, methyltransferases; SAH, S‐adenosylhomocysteine; SAHH, S‐adenosylhomocysteine hydrolase; SAM, S‐adenosylmethionine; SAMS, S‐adenosylmethionine synthetase; SHMT, serine hydroxymethyltransferase; THF, tetrahydrofolate.

MTR is a key enzyme in the one‐carbon cycle, utilizing methyl groups derived from 5‐methyltetrahydrofolate (5‐methyl THF) and aided by vitamin B_12_ to reconvert homocysteine (Hcy) to methionine. Therefore, we hypothesize that the benefits brought by the downregulation of MTR may be related to methionine metabolism. Methionine serves as the substrate of S‐adenosylmethionine (SAM), the primary methyl donor in organisms, and this reaction is catalyzed by S‐adenosylmethionine synthetase (SAMS) (Cantoni 1975). Both DR and metformin significantly reduce SAM levels, leading us to investigate whether the reduction of SAM accounts for the life‐prolonging effect. We used cycloleucine (cLEU), a competitive inhibitor of SAMS (Jani et al. 2008; Lombardini and Talalay 1971), to block SAMS and reduce SAM production, and then assessed its effects on rotifers (Figure 5a,b). We found that cLEU significantly extended the lifespan of rotifers, with a reproductive profile strikingly similar to DR‐ and metformin‐treated rotifers. cLEU treatment significantly reduced daily egg production while extending the reproductive period (Figure 5c–e). Instead, excessive supplementation of methionine or SAM abolished the lifespan‐extending effects of DR or metformin (Figure 5f,g). Overall, inhibiting methionine metabolism and reducing SAM synthesis can extend the lifespan of rotifers.

## 3 Discussion

In this study, we investigated the regulatory effects of metformin and DR on m6A at the whole‐organism level using rotifers, as well as the association between these effects and their antiaging properties. We identified the m6A‐MTR axis as a key posttranscriptional regulatory pathway for methionine homeostasis. Both metformin and DR target this pathway to reduce MTR expression and SAM production, thereby extending rotifer lifespan.

Many studies have provided compelling evidence that metformin can extend lifespan and attenuate several aging hallmarks by multiple pathways in models such as mouse, nematode, and cultured cells (Kulkarni et al. 2020; Martin‐Montalvo et al. 2013; Onken and Driscoll 2010; Slack et al. 2012; Smith Jr. et al. 2010). However, no evidence shows that metformin can extend the fruit flies' lifespan and even has toxic effects, though it can alleviate oxidative stress‐related DNA damage in the intestinal stem cells (Na et al. 2013; Slack et al. 2012). In nematode, Cabreiro et al. reported that the lifespan‐extending effect of metformin is indirectly mediated through changes in bacterial metabolism, the worms' food, whereas Xiao et al. found that metformin can act directly on the worms themselves (Cabreiro et al. 2013; Xiao et al. 2022). These interspecies and mechanistic differences prompted us to explore the effects of metformin in a different model organism. While *C. elegans* and *D. melanogaster* are the prominent invertebrate models for aging research, they do have certain limitations (Mack et al. 2018; Piper and Partridge 2018). They both belong to the Ecdysozoa and share a closer evolutionary relationship than previously believed, which narrows the scope for comparative aging studies (Austad 2009; Dunn et al. 2008; Edgecombe et al. 2011). Furthermore, they display unique nonaging life history stages in response to environmental stress, such as the “dauer” stage in worms and reproductive diapause in flies (Hu 2007; Larsen et al. 1995; Tatar et al. 2001). Humans lack these distinct life history stages, so antiaging interventions targeting pathways may not be directly applicable. Therefore, we selected an organism from Lophotrochozoa, the monogonont rotifer *B. asplanchnoidis*, which lacks metamorphosis or specialized life history stages that could confound aging‐related studies in response to environmental changes (Boell and Bucher 2008; Dunn et al. 2008). Additionally, rotifers are easy to cultivate, reproduce asexually, and have a short lifespan, making them a valuable addition for aging studies (Gribble and Mark Welch 2017; Gribble and Snell 2018; King 1969; Snell 2014).

Metformin acts directly on rotifers, extending their lifespan. Metformin reduced aging‐related biomarkers, decelerated the reproductive clock, and prolonged the reproduction period without impacting total egg production (Figure 1a–d). This result parallels findings in *C. elegans* , where metformin‐treated worms displayed delayed egg‐laying and showed higher progeny produced on metaphase of reproduction (Onken and Driscoll 2010). Metformin is often considered a mimetic of DR, inducing a transcriptional profile similar to that observed under DR conditions (Dhahbi et al. 2005). A previous study has shown that metformin cannot extend the lifespan of worms in the Eat‐2 DR model (Onken and Driscoll 2010), and similarly, our study also reveals that metformin does not prolong the lifespan of rotifers under DR condition (Figure 1g). These data indicate that the mechanisms of action of metformin may overlap with those of DR and may be conserved across different species. Furthermore, the resemblance in reproductive profile under DR further supports this hypothesis (Figure 1d,h).

m6A is one of the most abundant RNA modifications in eukaryotic RNA, dynamically changes as a posttranscriptional means of regulating and effecting cellular adaptation in response to specific stimuli, which has important roles in cell growth, embryo development (Villa et al. 2021; Zhao et al. 2017), neuronal activities (Worpenberg et al. 2021), sex determination, gametogenesis (Hongay and Orr‐Weaver 2011; Lence et al. 2016; Liu et al. 2023), and aging (Jiang et al. 2021; Min et al. 2018; Wu et al. 2023; Wu et al. 2020). Our previous work shows that rotifers have an m6A system that is more similar to that of vertebrates than the m6A systems of nematodes and fruit flies are to the vertebrates' m6A system, despite lacking m6A demethylases (Lence et al. 2017; Sendinc et al. 2020; Zhang et al. 2023). Using the m6A‐MeRIP‐seq technique, we identified approximately 4000 m6A peaks in the rotifer genome, significantly fewer than the tens of thousands of peaks observed in vertebrates, a result consistent with findings from yeast studies (Dominissini et al. 2012; Schwartz et al. 2013; Wang et al. 2014). The expression of m6A‐related enzymes in yeast and rotifers does not appear to be as broad as in vertebrates. In yeast, the m6A methylation program is confined to meiosis, while in rotifers, m6A enzymes are upregulated specifically during the reproductive stage (Zhang et al. 2023). This may explain the fewer m6A methylation sites in rotifers compared to vertebrates. Although most m6A peaks are quiescent, DR and metformin each specifically regulate hundreds of m6A peaks on transcripts, and the genes with differential peaks are highly enriched in transcriptional regulation‐related categories. A joint analysis of MeRIP‐seq and transcriptome identified two genes, MTR and PLIN2, coregulated by DR and metformin, showing significant changes in gene expression (Figure 3c). PLIN2 (Perilipin 2), an adipose differentiation‐related protein crucial for the formation and maintenance of lipid droplets (Doncheva et al. 2023; Xu et al. 2019), may account for the decreased fat deposition observed under DR and metformin treatment. The gene MTR, with its m6A modification specifically inhibited by DR and metformin, showed a reduced mRNA half‐life (Figure 3i). MTR connects the folate cycle with the methionine cycle, which together form the one‐carbon cycle, a universal metabolic process that activates and transfers one‐carbon units for various biosynthetic processes, including purine, thymidine synthesis, and methionine resynthesis (Ducker and Rabinowitz 2017; Yang and Vousden 2016).

Manipulating the one‐carbon metabolism pathway can promote geroprotection and extend healthy longevity, with methionine restriction being a key mechanism (Lee et al. 2016; Miller et al. 2005; Orentreich et al. 1993; Richie et al. 1994). Methionine restriction has been shown to extend lifespan across species, from yeast to vertebrates. However, methionine acts as a double‐edged sword in health and disease (Navik et al. 2021). Long‐term restriction of methionine leads to adverse effects such as stunted growth, decreased bone mass, and induced hyperhomocysteinemia (Elshorbagy et al. 2010; Huang et al. 2014; Parkhitko et al. 2019). Similarly, while moderate methionine supplementation is helpful for health, excessive intake may be harmful (Alzoubi et al. 2020; El‐Dessouki et al. 2016; Kumar et al. 2020; Li et al. 2020). Our results support this; continuous inhibition of methionine synthesis or excessive supplementation of methionine and its metabolite SAM shortens the lifespan of rotifers (Figure 4h–k & 5f,g). Therefore, regulation of methionine homeostasis is of great significance to health and longevity. Methionine is an essential amino acid that is obtained through diet and can only be remethylated from homocysteine (Hcy). This process involves either MTR, which requires 5‐methyltetrahydrofolate (5‐methyl THF) as a methyl donor and Vitamin‐B_12_ as a cofactor, or BHMT (betaine homocysteine methyltransferase), which requires betaine as a methyl donor. However, in most invertebrates, other than echinoderms, only the MTR‐mediated Hcy remethylation pathway is present (Delgado‐Reyes et al. 2001; Gadagkar et al. 2015). MTR has been reported to be regulated by a variety of mechanisms to maintain methionine homeostasis, including the alteration of mRNA splicing patterns, which are crucial for coordinating metabolism in the brain during development and aging (Phillips et al. 2013). In the placenta of women delivering preterm, the promoter region of MTR is hypermethylated at cytosine‐guanine (CpG) sites, leading to a lower protein level of MTR (Khot et al. 2017). Additionally, the activity of MTR can be enhanced posttranscriptionally by Vitamin‐B_12_ (Gulati et al. 1999). In this study, we uncovered that the stability of MTR transcripts can be regulated by m6A modification. Collectively, these findings suggest that MTR is not merely a housekeeping gene but is subject to extensive regulatory control in metabolism, disease, and aging processes.

Metformin has previously been reported to regulate the one‐carbon cycle in cancer cells, where it positively modulates SAH hydrolase activity, thereby altering genome‐wide DNA methylation (Zhong et al. 2017). Similarly, Cuyàs et al. demonstrated that metformin increases the one‐carbon flux to the methionine cycle and enhances the methylation capacity by inhibiting mitochondrial respiratory chain complex I (Cuyas et al. 2018). Notably, Cabreiro et al. inferred that metformin inhibits MTR in *E. coli* through the accumulation of 5‐methyl THF and the reduction of THF, a phenomenon known as “methyl trap” (Cabreiro et al. 2013). However, our findings suggest that metformin may directly inhibit MTR through m6A modification (Figure 5h), even though the RNA m6A pathway is not applicable in prokaryotes. Although molecular mechanisms may differ across species, the suppression of MTR expression or activity by metformin could be a conserved effect. Further experimental validation is needed to confirm this hypothesis. It should be noted that although RNA interference supported MTR's role in aging in this study, more rigorous experiments are needed to validate the statements proposed here. This is particularly crucial due to the current limitations of genetic tools for rotifers. To date, Gribble's team has made significant progress in the gene editing of rotifers (Feng et al. 2023). Future work should employ CRISPR‐based mutagenesis and rotifer‐optimized antibodies (for RIP‐qPCR and protein expression detection) to definitively verify m6A reader function and solidify causal mechanisms.

Disruption of epigenetic information is one of the hallmarks of aging, and induced epigenetic reprogramming is an attractive means to reverse aging (López‐Otín et al. 2013, 2023). DNA methylation reprogramming mediated by the Yamanaka factors can induce fibroblasts to become pluripotent stem cells (Takahashi and Yamanaka 2006), and enhance the regenerative capacity of neural axons, which decline with aging (Lu et al. 2020). Our results suggest that m6A methylation reprogramming of mRNA also has a positive effect on intervening in aging and extending lifespan by regulating gene transcription, potentially offering a novel perspective for antiaging therapies.

In summary, this work advances our understanding of the relationship between mRNA epigenetic information alteration, methionine homeostasis, and aging. It also provides new insights into the antiaging effects of metformin and DR, highlighting rotifers as a valuable complement to canonical invertebrate models in geroscience and experimental gerontology.

## 4 Materials and Methods

### 4.1 Rotifer and Algae Culture

*B. asplanchnoidis* was cultured in artificial seawater (ASW) with 30 parts per thousand (ppt) concentration prepared by concentrated sea salt (Blue Grand Star, China). This clonal strain was kept in an illumination incubator with 4000 lx light intensity, 12:12 h light: dark photoperiod at 25°C. The rotifer culture method is according to Snell's method with minor modifications (Snell et al. 2012).

The algae *Chlorella vulgaris* was cultured in F/2 medium (Guillard 1975) at 25°C with 24 h light exposure (4000 lx) and collected at the exponential growth stage by centrifugation at 5000 rpm for 5 min. Rotifers were fed with concentrated *C. vulgaris* at 3 × 10^6^ cells/mL once a day.

### 4.2 Synchronization of Rotifers

In order to eliminate the interference caused by the different development stages of rotifers in the experiment, we need to synchronize their development. Amictic eggs were collected in a 1.5 mL centrifuge tube by forcefully pipetting adult rotifers through micropipette tips to separate the eggs from the mothers (Figure S8) (Feng et al. 2023). The eggs were then transferred to ASW containing *C. vulgaris* as a food source and left to hatch for 4 h. The age‐synchronized neonates were employed in subsequent experiments. Except for the life table experiment, healthy 6‐day‐old rotifers were used in all other experiments.

### 4.3 Life Table Experiment

Full cohort life tables were performed according to Snell's method with minor modifications (Snell and Johnston 2014). Each neonate was placed in a separate well of a 24‐well plate, with 1 mL of culture medium per well. The medium contained 3 × 10^6^ cells/mL *C. vulgaris* diluted in 30 ppt ASW, and 20 μM FUdR (2′‐Deoxy‐5‐fluorouridine, Aladdin‐F110732, Shanghai, China). FUdR was used to inhibit the hatching of newborn eggs (Snell et al. 2012). Each treatment consisted of 48 individuals. Animals were transferred to fresh medium every 3 days under the aforementioned culture conditions. Daily inspections were made on the plates to monitor animal survival and progeny until all individuals were dead.

### 4.4 Staining for Lipid Content, SA‐β‐Gal Activity and Assessing Ovarian Development

The rotifers were fixed in 4% paraformaldehyde at room temperature for 1 h and then gently washed with fresh ASW. To visualize lipid accumulation, we employed Oil Red O, a lipid‐soluble azo dye and a potent lipid‐soluble reagent used for fat staining. It can specifically stain neutral lipids, such as triglycerides, within cells or tissues. The rotifers were stained with Oil Red O solution (Beyotime‐C0158S, Shanghai, China) at room temperature for 30 min. Next, to assess cellular senescence, we evaluated the activity of SA‐β‐gal (senescence‐associated‐β‐galactosidase), a widely recognized biomarker for senescent cells (Hernandez‐Segura et al. 2018). SA‐β‐Gal staining was performed using a commercial kit (Beyotime‐C0602, Shanghai, China). The fixed rotifers were stained with the SA‐β‐Gal staining solution at 37°C overnight. For staining rotifer tissue, methylene blue (Aladdin‐M196500, Shanghai, China) was used. It interacts specifically with intracellular molecules like nucleic acids and proteins to achieve the staining effect. The fixed rotifers were transferred to a solution containing 0.02% methylene blue (in ASW) and stained at room temperature for about 1 h. After staining, images were captured at 20 × magnification using a BX43 microscope with a DP28 digital camera (Olympus, Japan). All images were analyzed via Fiji software (National Institutes of Health, USA) (Schindelin et al. 2012).

### 4.5 m6A Dot Blot Assay

Dot blot was performed as previously reported (Nagarajan et al. 2019; Shen et al. 2017). Briefly, total RNA was extracted using a commercial kit (TIANGEN‐DP451, Beijing, China), and mRNA was captured with the BeyoMag mRNA Purification Kit (Beyotime‐R0071S, Shanghai, China). The mRNA was hybridized to positively charged nylon membranes (Beyotime‐FFN13, Shanghai, China) using a UV cross‐linker (Stratalinker 2400 UV, USA). To minimize nonspecific cross‐reactivity, the membranes were blocked for 2 h at room temperature in a blocking buffer consisting of 5% nonfat milk with TBST (TBS with Tween20). The m6A‐specific primary antibody (Beyotime‐AF7407, Shanghai, China) was diluted in TBST (1: 2500) and incubated with the blots for 12 h at 4°C. The membranes were washed three times for 10 min each in TBST before being incubated with antirabbit IgG secondary antibodies (1: 2500). Blots were detected using an ECL chemiluminescence assay (BOSTER‐AR1190, Wuhan, China) according to the manufacturer's instructions. The images were obtained using a Tanon system with accompanying software (Shanghai, China) and quantified via Fiji software (National Institutes of Health, USA) (Schindelin et al. 2012). Methylene blue (MB) staining was used as a loading control.

### 4.6 S‐Adenosylmethionine ELISA

SAM levels of rotifers were measured using the ELISA kit (Cloud Clone‐CEG414Ge, Pan‐species) following the manufacturer's protocol. After appropriate treatment, about 5000 rotifers were homogenized in 500 μL ice‐cold phosphate‐buffered saline (PBS) solution containing 1 mM PMSF and a protease inhibitor cocktail (Beyotime‐ST507 & P1005, Shanghai) using a grinding apparatus (Jingxin, Shanghai). The homogenate was centrifuged at 10000 g for 5 min in 4°C, and the supernatant was immediately proceeded for ELISA. The 450 nm OD readings of the samples were plotted on a standard curve obtained using the kit's SAM‐BSA standards. This allowed us to derive SAM levels in the test samples. Furthermore, the SAM levels were normalized to the protein amount loaded for comparative analysis. Protein concentration was measured by the BCA method (Walker 1994).

### 4.7 m6A Methylated RNA Immunoprecipitation Assay

The procedure of m6A immunoprecipitation (m6A‐MeRIP) was performed as previously reported methods (Dominissini et al. 2012). In brief, mRNA was purified by two rounds of purification from 50 μg total RNA using Dynabeads Oligo (dT) 25–61,005 (Thermo Fisher, CA, USA). Then the purified mRNA was fragmented into small pieces using the Magnesium RNA Fragmentation Module (NEB‐E6150, USA) under 86°C for 7 min. The cleaved RNA fragments were incubated for 2 h at 4°C with m6A‐specific antibody (Synaptic Systems‐202,003, Germany) in IP buffer (50 mM Tris–HCl, 750 mM NaCl and 0.5% Igepal CA‐630). The IP RNA was reverse‐transcribed to cDNA by SuperScript II Reverse Transcriptase (Invitrogen‐1,896,649, USA), which was next used to synthesize U‐labeled second‐stranded DNAs with DNA polymerase I (NEB‐M0209, USA), RNase H (NEB‐M0297, USA) and dUTP Solution (Thermo Fisher‐R0133, USA). An A‐base was added to the blunt ends of each strand, preparing for ligation to the indexed adapters. Dual‐index adapters were ligated to the fragments, and size selection was performed with AMPure XP beads (Beckman Coulter‐A63880, USA). After the heat‐labile UDG enzyme (NEB‐M0280, USA) treatment of the U‐labeled second‐stranded DNAs, the ligated products were amplified with PCR under the following conditions: initial denaturation at 95°C for 3 min; 8 cycles of denaturation at 98°C for 15 s, annealing at 60°C for 15 s, and extension at 72°C for 30 s; and then final extension at 72°C for 5 min. At last, we performed paired‐end sequencing on an Illumina Novaseq 6000 platform following the vendor's recommended protocol.

### 4.8 A Single‐Base Detection of Locus‐Specific m6A

Methods to detect specific m6A modifications of single transcript of interest are crucial for studies of m6A biological functions. Xiao et al. have developed a method called “SELECT”, which is based on the characteristic of m6A inhibiting DNA polymerase elongation and ligase ligation, and is highly sensitive as well as radiolabeling‐free (Xiao et al. 2018). In brief, the differentially modified fragments and sites were identified based on the MeRIP‐seq data, and then a pair of specific probes was designed that complemented the fragments but left a gap at the modified site. Subsequently, the total RNA was mixed with the probes (0.04 μM), dNTPs (5 μM), and 17 μL of 1 × CutSmart buffer (NEB, USA). The RNA and probes were annealed by incubating mixture at a temperature gradient: 90°C for 1 min, 80°C for 1 min, 70°C for 1 min, 60°C for 1 min, 50°C for 1 min, and then 40°C for 6 min. Subsequently, 3 μL of a mixture containing 0.01 U Bst 2.0 DNA polymerase (Takara‐RR380A, Japan), 0.5 U Splint R ligase (Beyotime‐D7018S, Shanghai, China) and 10 nmol ATP (Beyotime‐D7378, Shanghai, China) was added to the former mixture to reach a final volume of 20 μL. The final reaction mixture was incubated at 40°C for 20 min, denatured at 80°C for 20 min and then kept at 4°C. Afterwards, 2 μL of the final reaction mixture was used as template to perform qPCR at the following condition: Predenaturation at 95°C for 60 s, 40 cycles of denaturation at 95°C for 15 s, and annealing and extension at 60°C for 60 s. Melting curve steps: 95°C for 15 s, 60°C for 60 s, 95°C for 15 s and continuous (Roche, Light Cycler 96, Switzerland). The qPCR cycles for sites beyond ±6 (A‐10, A + 13) bases of the modification site are used to normalize the amount of RNA input. Data were collected as threshold cycle (CT) values and the ΔΔCt method was used to calculate fold changes (Livak and Schmittgen 2001). All primers used in this study were listed in Table S1.

### 4.9 RNA Stability Analysis

Assay for RNA stability analysis was performed as previously described with some modifications (Huang et al. 2018; Wang et al. 2014). Rotifers were cultured in a 50‐mL beaker with 15 mL ASW. Actinomycin D (ACTD) was used to inhibit the transcription process with a final concentration of 15 μg/mL (Aladdin, A113142, Shanghai, China). Rotifers were collected at three time points (0, 4, 8 h) after the addition of ACTD, and total RNA was extracted (TIANGEN, DP‐451, Beijing, China). Then the RNA was reverse transcribed using a First Strand cDNA Synthesis Kit with a gDNA eraser (Beyotime‐D7170M, Shanghai, China). RT‐qPCR was performed as above and ACTB was used as a loading control for normalization. For each RNA transcript of interest, a semilog graph was plotted, and its RNA half‐life (t_1/2_) was determined in each condition tested. The degradation rate (k) was estimated by the equation **log**_**2**_ (Ct/C0) = −k*t, where t is transcription inhibition time (h), Ct and C0 represent mRNA quantity at time t and time 0, respectively (Chen et al. 2008). Two *k* values were calculated: time 4 h versus time 0 h and time 8 h versus time 0 h. The final K_deacy_ was calculated by using the average of k‐4 h and k‐8 h. RNA half‐life (t_1/2_) was estimated by the equation **t**_**1/2**_ **= ln2/K**_**deacy**_ (Chen et al. 2008).

### 4.10 RNA Interference (RNAi) of Rotifer

As described in our previous work, RNAi in rotifers is achieved by the feeding method (Zhang et al. 2024). In brief, we used a plasmid L4440 with two reverse T7 promoters and an RNase III‐deficient *E. coli* strain, HT115 (DE3). We subcloned the CDS sequence of the target gene between the two T7 promoters of L4440 and verified it by sequencing. Subsequently, the recombinant plasmid (XXX‐L4440) was transformed into HT115. DE3 *E. coli* , which has been integrated T7 RNA polymerase gene, promising the production of dsRNA from the DNA fragment cloned between the two T7 promoters under induction with IPTG (isopropyl‐β‐D‐thiogalactoside). Due to the RNase deficiency in HT115, dsRNA can be stably maintained within the bacterial cells.

Bacteria carrying the recombinant plasmid were added into fresh LB medium and cultured at 37°C with shaking at 200 rpm until the optical density (OD_600_) reached approximately 0.6. Subsequently, IPTG was added to a final concentration of 0.5 mM to induce plasmid transcription. After 8 h of induction, the bacteria were harvested by centrifugation at 5000 rpm for 10 min, resuspended in ddH_2_O, and stored at 4°C. Total RNA was extracted from the induced bacteria and subsequently mixed with RNase A in a high‐salt concentration environment (NaCl ≥ 300 mM) to digest the single‐stranded RNA. This treatment allowed for the assessment of dsRNA induction efficiency via 1% agarose gel electrophoresis.

Approximately 5000 age‐synchronized rotifers were carefully transferred into a 50 mL beaker containing culture medium. The medium was composed of 3 × 10^6^ cells/mL of *C. vulgaris* suspended in 30 ppt ASW and supplemented with 20 μM 5′‐Deoxy‐5‐fluorouridine (FUdR, Aladdin‐F110732, Shanghai, China), which inhibits the hatching of newly born eggs (Snell et al. 2012). For the RNAi treatment, induced bacteria were added to the medium at a volume equal to one‐tenth of the total medium volume, resulting in a concentration of approximately 1 × 10^7^ cells/mL. The RNAi treatment duration was 48 h, during which the culture medium was refreshed daily. The efficiency of RNAi was assessed using qPCR, with the empty vector serving as the positive control.

### 4.11 Statistical Analysis

Statistical analysis was conducted by GraphPad Prism Software. The Shapiro–Wilk test or Kolmogorov–Smirnov test was used to assess normality and lognormality of the data. The comparisons between groups were analyzed by one‐way ANOVA, and a multiple‐comparison Tukey's test was used to determine significant differences between each group. If the data did not follow a normal distribution, the Kruskal–Wallis test followed by Dunn's multiple comparisons test was used. Student's *t*‐test was applied when two data sets were compared. Survival curve analysis was conducted by Log‐rank test. Data were presented as mean ± SD. *p*‐values < 0.05 were considered statistically significant.

## Author Contributions

Yu Zhang: conceptualization, original draft preparation, funding acquisition. Xiaojie Liu: review and editing, visualization. Hairong Lian and Yanchao Chai: methodology. Yang Zhou and Dongqi Kan: formal analysis. Jilong Ren and Cui Han: rotifer and algae culture. Jiaxin Yang: supervision, funding acquisition.

## Conflicts of Interest

The authors declare no conflicts of interest.

## Supporting information


Figure S1.

Data S1.

Data S2.

## Data Availability

The data that support the findings of this study are available from the corresponding author upon reasonable request.
