# Supplementary material for: Metformin and Dietary Restriction Counteract Aging via Reducing m6A–Dependent Stabilization of Methionine Synthase mRNA in Brachionus asplanchnoidis (Rotifera)
Source: Aging Cell. 2025 May 27;24(8):e70113. doi: 10.1111/acel.70113 (PMC12341819; doi:10.1111/acel.70113)
Supplement: Supplementary file 1 — Figure S1. [file ACEL-24-e70113-s002.docx]

Fig. S1 The pie chart shows the proportion of m6A peaks on the five major functional elements of genes (5’ UTR, start codon, CDS, stop codon, 3’ UTR) in each sample group.

Fig. S2 m6A motif identified in control, DR-, and metformin-treated rotifers. m6A modifications were typically located in a consensus ‘AGACY’ motif (Y: U/C), which is similar to the ‘RRACH’ motif (R: A/G; H: U/A/C) reported in vertebrates.

Fig. S3 Go analysis of the m6A-modified genes **shared** by control, DR-, and metformin-treated rotifers.

Fig. S4 Go analysis of the **control-specific** m6A-modified genes in rotifers.

Fig. S5 Go analysis of the **DR-specific** m6A-modified genes in rotifers.

Fig. S6 Go analysis of the **metformin-specific** m6A-modified genes in rotifers.

Fig. S7 Go analysis of differentially peaked mRNAs in DR and metformin-treated rotifers.

Fig. S8 Schematic diagram illustrating the step-by-step workflow for the synchronization of rotifers.

Table S1

Primers used in this work are as follows:

| Gene name | Application | Forward | Reverse |
| --- | --- | --- | --- |
| METTL3 | qPCR | TGGCTGAAGTCAGAGCAACT | AAAAGCCTAACGCCGTCCAA |
| METTL14 | qPCR | ATGAAGGCTGTGCTGATAA | GTCAACCATCCGTGTCTT |
| WTAP | qPCR | ATGTCGGAAGTTGCTCACCG | TATCGTGGCTTGCATTGCATC |
| Virma | qPCR | GTGGTCGACCTGATCGTGTT | GATTTTGAGCAGCAGTCGGC |
| MTR | qPCR | TGTGGCAACTCAGAGGCAAA | TGTCAGCGTTGCATTTGTGG |
| IGF2BP | qPCR | GTCATTCAGGGTTCGCCAGA | GACGGCACTTGTATTTCGGC |
| β-actin | qPCR | ACTGCTATGTTGGCGACGAA | CTGGAGCCACTCTCAACTCG |
| MTR-A-10 | SELECT | tagccagtaccgtagtgcgtg  TTTTGGCGTGTTTGGACAGC | 5’PHO-ATTGGGCGTACACGAGCATT  cagaggctgagtcgctgcat |
| MTR-A | SELECT | tagccagtaccgtagtgcgtg  CTCGGTCTTTTTGGCGTG | 5’PHO-TTGGACAGCGATTGGGC  cagaggctgagtcgctgcat |
| MTR-A+13 | SELECT | tagccagtaccgtagtgcgtg  ACACTTCGTCCGCCTCGG | 5’PHO-CTTTTTGGCGTGTTTGGACA  cagaggctgagtcgctgcat |
| Adapter | SELECT-qPCR | TAGCCAGTACCGTAGTGCGTG | ATGCAGCGACTCAGCCTCTG |
| IGF2BP | RNAi | **ggatcc**ATGCTACAAACACAAGACGAACAAA | **aagctt**GTTATAACACGCTCAACATTCATAC |
| MTR | RNAi | **ggatcc**ATGAGTTTGCACGAAGAATTAAGAAA | **aagctt**GACCACGACATTGTTCAGTGTACG |

The lowercase parts of the SELECT primers are universal adapters used for subsequent qPCR validation. 5’PHO indicates phosphorylation modification at 5’ end of the primers. The bold parts represent restriction enzyme cutting sites, ‘ggatcc’ corresponds to BamHⅠ, and ‘aagctt’ corresponds to HindⅢ.
